# Strangulated Epigastric Hernia: A Rare Occurrence

**DOI:** 10.7759/cureus.14038

**Published:** 2021-03-22

**Authors:** Sri Hari Priya Vemulakonda, Souradeep Dutta, Ankit Jain, Abhinaya Reddy, Vishnu Prasad Nelamangala Ramakrishnaiah

**Affiliations:** 1 Surgery, Jawaharlal Institute of Postgraduate Medical Education and Research, Puducherry, IND

**Keywords:** epigastric hernia, strangulated, incarcerated, linea alba hernia

## Abstract

An epigastric hernia is a rare type of abdominal hernia, described in the literature mostly as small, containing only preperitoneal fat. A large true epigastric hernia with herniation of the abdominal viscera is even rarer. Only a few case reports have given an account of strangulation in such an epigastric hernia. This case report describes a middle-aged, morbidly obese man with a big epigastric hernia presenting with incarceration and acute abdominal pain. Emergency surgical exploration revealed a 7 cm midline defect in the rectus sheath and a 30 cm segment of the jejunum and a 6 cm segment of the transverse colon were gangrenous. The gangrenous bowel segments were resected, and an end-to-end jejuno-jejunal and colo-colic anastomosis were done. The patient had an uneventful postoperative recovery.

## Introduction

A hernia is the protrusion of a viscus or part of a viscus through a defect in its containing cavity wall. The common types of external abdominal hernias are inguinal (75%), umbilical (15%), femoral (8.5%), and incisional (3.5-11%). Epigastric hernias account for around 1.6-3.6% of all abdominal hernias and 0.5-5% of all operated abdominal hernias [1]. A hernia is called strangulated when the blood supply of its contents is compromised. The precipitating cause of strangulation is usually unknown but is presumably some event that forces more abdominal viscera into the sac that can be easily returned. A strangulated epigastric hernia is very rare and is associated with significant morbidity and mortality. We report a rare case of a strangulated epigastric hernia.

## Case presentation

A 42-year-old gentleman with severe obesity (weight 130 kg, BMI 45.0 kg/m^2^) presented with epigastric swelling for one year which was initially reducible but became irreducible for one day. It was associated with sudden onset of upper abdomen pain with abdominal distension and non-bilious vomiting for one day.

**Figure 1:**
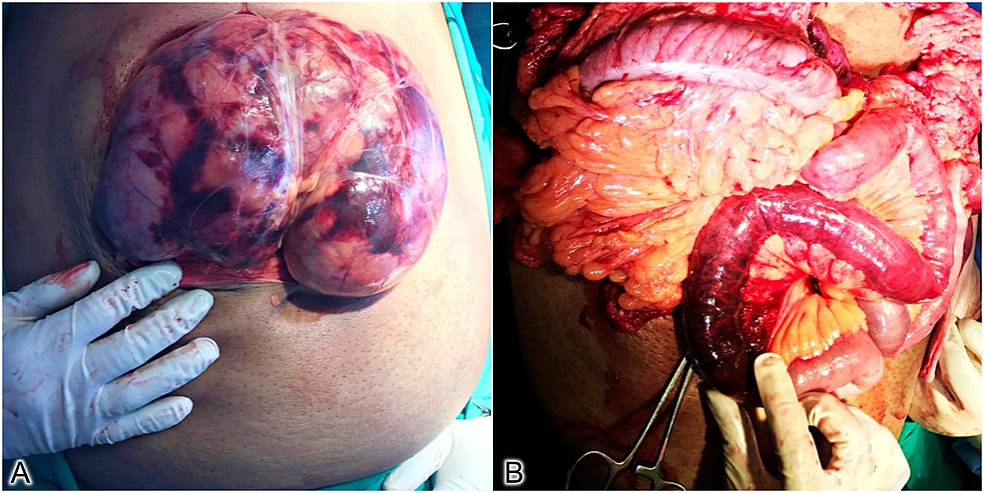
Intraoperative photos. (A) Herniated bowel with peritoneal sac. (B) Gangrenous bowel segment.

At presentation, he had tachycardia (pulse rate 110/min) but was maintaining blood pressure. On examination, a 10x15 cm tender irreducible swelling was present in the epigastrium. The skin over the swelling was normal. The rest of the abdomen was soft, but bowel sounds were absent. Ultrasonography (USG) of the abdomen revealed a supra-umbilical midline defect in the anterior abdominal wall. There was herniation of bowel loops and omentum, showing decreased vascularity and absent peristalsis. He was taken up for emergency laparotomy. Intra-operatively, a 7 cm midline defect was present in the rectus sheath. A hernial sac containing a 30 cm gangrenous segment of the jejunum (210 cm from the duodenojejunal flexure and 160 cm from the ileocecal junction) and a gangrenous segment of the transverse colon for a length of 6 cm was noted (Figure 1).

The gangrenous bowel segment was resected, and an end-to-end jejuno-jejunal and colo-colic anastomosis were done. He had an uneventful postoperative recovery and was discharged on postoperative day 10.

## Discussion

Any protrusion of tissue through a defect in the linea alba between the xiphoid process and the umbilicus is designated an epigastric hernia [2]. Most of these hernias occur in the immediate three inches above the umbilicus, while those near the xiphoid are exceedingly rare [3]. Epigastric hernias are more common in middle-aged men and obese patients [4]. Risk factors for an epigastric hernia include uncoordinated vigorous synchronous contractions of the diaphragm like coughing, heavy weightlifting, and athletic training.

The oldest theory for epigastric hernia is the perforating vessel theory by Moschowitz. The preperitoneal fat herniates through the defect for the perforator vessels in the rectus, forming an epigastric hernia [5]. This might lead to the protrusion of preperitoneal fat and the intra-abdominal contents through the linea alba. As per his observations, a true epigastric hernia with a peritoneal sac was a rarity, and most cases had only herniation of preperitoneal fat without a sac [6]. Askar proposed a new anatomical basis of the pathogenesis. He suggested a single midline pattern of aponeurotic decussation rather than a triple decussation was responsible for the weakness, leading to herniation [7]. Multiple studies and reports have supported and challenged these observations, but none have been proved convincingly [8,9]. A true epigastric hernia with proper sac and bowel contents is a rare occurrence, and strangulation of bowel contents is even rarer, reported only by a few case reports worldwide [3,10,11].

Epigastric hernias are generally small, containing only preperitoneal fat. Therefore, patients present with periodical sharp localized pain in the epigastric region, often associated with dyspepsia and nausea without any relation to food intake [6]. Clinical diagnosis of a strangulated epigastric hernia is difficult unless there is high suspicion. Gangrene may occur as early as six hours after the onset of strangulation [12]. Patients usually present with a long-term epigastric swelling with sudden onset irreducibility, severe pain, and abdominal distension. Clinically they worsen very quickly. Although it is a clinical diagnosis based on sudden onset irreducibility of a previous reducible swelling, ultrasonography (USG) of the abdominal wall often clinches the diagnosis. USG helps identify the size of the defect, contents of the sac, and their vascularity.

Epigastric hernias, similar to other hernias, need surgery because of their symptomatic nature. Early surgical intervention of an obstructed epigastric hernia is crucial as delay can result in strangulation and the need for bowel resection with prolonged recovery and increased complication rate. If preperitoneal fat or intraperitoneal hernial contents are healthy, they can be reduced and defects in the rectus sheath can be repaired. The repair has to be augmented with mesh placement [13]. However, if the contents are gangrenous, an urgent exploratory laparotomy with resection of gangrenous segment and anastomosis or ileostomy, depending on patients’ general condition, is the treatment of choice. Strangulated hernia increases the risks in emergency hernia repair, leading to an increased incidence of surgical site contamination and recurrence [14].

## Conclusions

A true epigastric hernia with a peritoneal sac and bowel contents is a rare occurrence. Therefore, a high index of suspicion for strangulation should be kept in cases of a big epigastric hernia with acute abdominal pain and recent-onset irreducibility. Early diagnosis and surgical management are the keys to avoiding bowel resection with prolonged recovery and increased complication rate.
